# Antiproliferative and Carbonic Anhydrase II Inhibitory Potential of Chemical Constituents from *Lycium shawii* and *Aloe vera*: Evidence from In Silico Target Fishing and In Vitro Testing

**DOI:** 10.3390/ph13050094

**Published:** 2020-05-13

**Authors:** Najeeb Ur Rehman, Sobia Ahsan Halim, Majid Khan, Hidayat Hussain, Husain Yar Khan, Ajmal Khan, Ghulam Abbas, Kashif Rafiq, Ahmed Al-Harrasi

**Affiliations:** 1 Natural & Medical Sciences Research Center, University of Nizwa, P.O Box 33, 616 Birkat Al Mauz, Nizwa, Sultanate of Oman; najeeb@unizwa.edu.om (N.U.R.); sobia_halim@unizwa.edu.om (S.A.H.); majid.khan@unizwa.edu.om (M.K.); hussainchem3@gmail.com (H.H.); husainyar@gmail.com (H.Y.K.); ajmalkhan@unizwa.edu.om (A.K.); kashifrafiq609@gmail.com (K.R.); 2 HEJ Research Institute of Chemistry, University of Karachi, Karachi 75270, Pakistan; 3 Department of Bioorganic Chemistry, Leibniz Institute of Plant Biochemistry, 06120 Halle, Germany; 4 Department of Biological Sciences and Chemistry, University of Nizwa, P.O Box 33, 616 Birkat Al Mauz, Nizwa, Sultanate of Oman; abbashej@unizwa.edu.om; 5 Department of Chemistry, Abdul Wali Khan University Mardan, Mardan 23200, Pakistan

**Keywords:** *Lycium shawii* Roem. & Schult, *Aloe vera* (L.) BURM. F., antiproliferative, antioxidant, pharmacophore modeling, inverse molecular docking, carbonic anhydrase II

## Abstract

*Lycium shawii* Roem. & Schult and resin of *Aloe vera* (L.) BURM. F. are commonly used in Omani traditional medication against various ailments. Herein, their antiproliferative and antioxidant potential was explored. Bioassay-guided fractionation of the methanol extract of both plants led to the isolation of 14 known compounds, viz., **1**–**9** from *L. shawii* and **10**–**20** from *A. vera.* Their structures were confirmed by combined spectroscopic techniques including 1D (^1^H and ^13^C) and 2D (HMBC, HSQC, COSY) nuclear magnetic resonance (NMR), and electrospray ionization-mass spectrometry (ESI-MS). The cytotoxic potential of isolates was tested against the triple-negative breast cancer cell line (MDA-MB-231). Compound **5** exhibited excellent antiproliferative activity in a range of 31 μM, followed by compounds **1**–**3**, **7**, and **12**, which depicted IC_50_ values in the range of 35–60 μM, while **8**, **6**, and **9** also demonstrated IC_50_ values >72 μM. Subsequently, in silico target fishing was applied to predict the most potential cellular drug targets of the active compounds, using pharmacophore modeling and inverse molecular docking approach. The extensive in silico analysis suggests that our compounds may target carbonic anhydrase II (CA-II) to exert their anticancer activities. When tested on CA-II, compounds **5** (IC_50_ = 14.4 µM), **12** (IC_50_ = 23.3), and **2** (IC_50_ = 24.4 µM) showed excellent biological activities in vitro. Additionally, the ethyl acetate fraction of both plants showed promising antioxidant activity. Among the isolated compounds, **4** possesses the highest antioxidant (55 μM) activity followed by **14** (241 μM). The results indicated that compound **4** can be a promising candidate for antioxidant drugs, while compound **5** is a potential candidate for anticancer drugs.

## 1. Introduction

Cancer is one of the most dreadful diseases in the whole world. Due to this disease, nearly 8.2 million people died in 2012 and approximately 14.1 million new cases were reported [1]. Despite having advanced treatments in the world, the number of deaths is dramatically increasing annually. Secondary metabolites, obtained through bioassay guided isolation from medicinal plants, or their derivatives are major ingredients of anticancer drugs. Over 150 natural-product-derived drugs came on the market between 1981 and 2014 [2]. Evidence from clinical trials, in vivo animal studies, and tissue culture suggested that more than 20,000 natural products or secondary metabolites have the potential ability to reduce the development and severity of certain types of cancers [3]. The use of natural constituents for drug discovery is increasing day by day worldwide with growing interest in the development of healthcare systems [4].

Over the past few decades, with the nonstop developments in chemotherapy, the improvements in early detection, and the advances of personalized therapy, the survival rates of patients having breast cancer (BC) have dramatically increased. However, despite this development, BC still remains the foremost cause of cancer-related death for women worldwide [5,6], with 535,000 deaths (2016) in 195 countries across the world [7,8], and significant clinical challenges [9]. BC can be subdivided into four main molecular subtypes (luminal B, luminal A, triple-negative (TN), and Her2-enriched) on the basis of the expression of the progesterone receptor (PR), epidermal growth factor receptor 2 (ERBB2, also called HER2), and estrogen receptor (ER) [10]. Triple-negative breast cancer (TNBC), the most intense, critical, and fast-growing type of BC, does not express progesterone receptors (PR) or estrogen receptors (ER), and lacks human epidermal growth factor receptor 2 (HER2) [11,12,13,14]. Due to the lack of these receptors, common treatments (hormone therapy and drugs) that target ER, PR, and HER-2 are ineffective, thus, treatment options for TNBC are limited. In this scenario, cytotoxic chemotherapy is the mainstay treatment option. Although TNBC tends to respond well to initial chemotherapy in the earlier stages, it tends to recur more frequently than other breast cancers [15]. The treatment of TNBC (highly metastatic subtype) is still challenging due to the deficiency of targeted therapy. Therefore, new treatment modalities are urgently required to save human lives [16,17].

Carbonic anhydrases (CAs, EC 4.2.1.1) are zinc-containing metalloproteinases which reversibly catalyze the conversion of CO_2_ to bicarbonate (HCO_3_-) ions [18]. The control of acid–base homeostasis is crucial for normal cell growth and probably plays an important role in tumorigenesis [19,20]. The extracellular pH in tumors is more acidic than the intracellular pH [21,22]. To create the pH gradient between the outside and inside cell compartments, tumor cells increase ion transport proteins and CA enzymes [19,21,22,23,24]. Enzymatically active CA isozymes (11) were identified in mammals including four cytosolic (CA I, II, III, and VII); two mitochondrial (CA VA and VB); one secretory (CA VI); and four membrane-associated (CA IV, IX, XII, and XIV) [19]. The CA II is expressed in malignant brain tumors [25], renal cancer cell lines, and gastric and pancreatic carcinomas [26,27,28,29]. CA II inhibitors can be used as an adjunct to chemotherapy for such cancers.

A number of medicinal plants are reported to possess anticancer and antioxidant properties due to the presence of phenols, flavonoids, flavonoid glycosides, and tannins. Antioxidants are those constituents which delay, prevent, or remove oxidative stress, and, in turn, oxidative damage to a target cell caused by free radicals [30]. Their important role is to prevent damage to cellular constituents arising as a result of chemical and biological reactions involving free radicals [31]. The synthetic antioxidants are easier to process than natural antioxidants. However, limitations in the practice of synthetic antioxidants have been prescribed because of toxicity and health risks [32]. Hence, synthetic antioxidants can be replaced by safer natural antioxidants [33].

*Lycium shawii* Roem. & Schult, a native plant of the Arabian Peninsula including Oman, Egypt, Bahrain, Saudi Arabia, United Arab Emirates, Kuwait, and Qatar [34], has shown hypotensive, spermatatoxic, antiplasmodial, antioxidant, and antidiabetic potential. Moreover, it is commonly used in traditional medicines to treat jaundice, stomach, mouth sores, and coughs [35]. *A. vera* (L.) BURM. F. is currently exploited for the treatment of arthritis, Crohn’s disease, ulcerative colitis, asthma, ulcers, sores, cold, acne, and burns [36]. Traditionally, *Aloe vera* resin is used in the management of diabetes, obesity, and other infectious diseases [37]. Previous findings investigated beneficial effects of different parts (leaves and fruits) of *L. shawii* but, to the best of our knowledge, there is no report on the antioxidant activity of *L. shawii* stem extracts. The biological activities of the above plants encouraged our group to further examine the phytochemical composition and biological activities of *L. shawii* and *A. vera* resin which led to the isolation of 20 known compounds. The isolated compounds were scrutinized for their cytotoxic and antioxidant behavior, and the anticancer mechanism of active compounds was predicted by in silico target fishing.

## 2. Results and Discussion

### 2.1. Phytochemical Investigation

**Figure 1:**
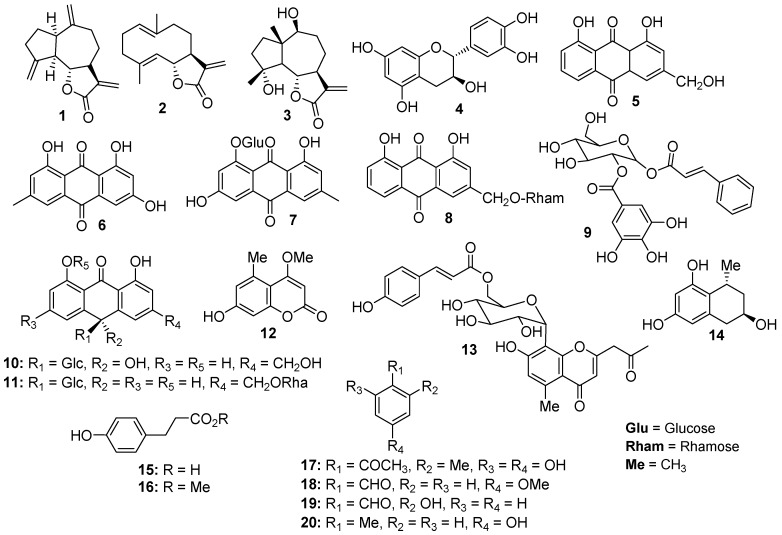
Structures of the compounds **1**–**9** isolated from *L. shawii* and **10**–**20** isolated from *A. vera* resin.

Phytochemical analysis of *L. shawii* led to the isolation of 9 compounds: dehydrocostus lactone (**1**), costunolide (**2**), lyciumate (**3**), catechin (**4**), aloe emodin (**5**), emodin (**6**), emodin-8-*O*-*β*-*D*-glucoside (**7**), aloe emodine 11-*O*-rhamnoside (**8**), and lyciumaside (**9**) [35]. Similarly, phytochemical investigation of *A. vera* resin provided 11 compounds including 10-hydroxy aloin A (**10**) [38], aloinoside B (**11**) [39], 7-demethylsiderin (**12**) [40], 6′-*O*-coumaroylaloesin (**13**) [41], feroxidin (**14**) [42], 3-(4-hydroxyphenyl)propanoic acid (**15**), methyl 3-(4-hydroxyphenyl)propionate (**16**) [43], 1-(2,4-dihydroxy6-methylphenyl)ethanone (**17**) [44], *p*-anisaldehyde (**18**), salicylaldehyde (**19**) [45], and *p*-cresol (**20**) [46]. All structures of the compounds were confirmed by combined spectroscopic techniques including 1D (^1^H and ^13^C) and 2D (HMBC, HSQC, COSY) nuclear magnetic resonance (NMR), and electrospray ionization-mass spectrometry (ESI-MS) (Figure S1). The structures of the compounds are shown in Figure 1.

### 2.2. Cytotoxic Activity

**Figure 2:**
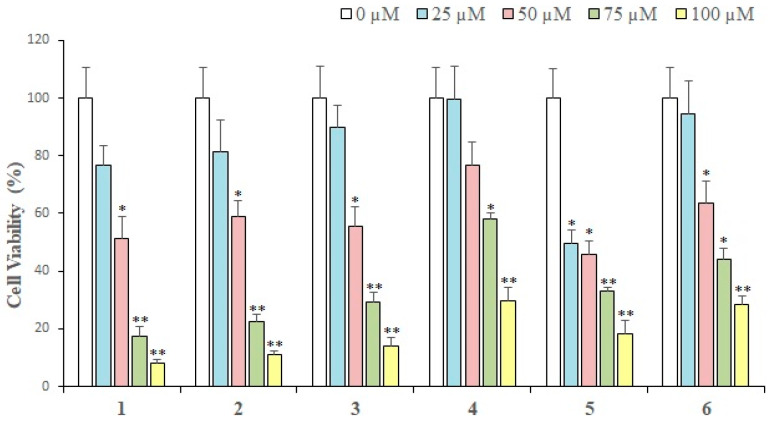
The cytotoxic effects of compounds **1**–**6** (isolated from *L. shawii*) on MDA-MB-231 breast cancer cells. All results are expressed as mean ± SEM. * *p* < 0.05 and ** *p* < 0.01, compared to respective untreated controls.

**Figure 3:**
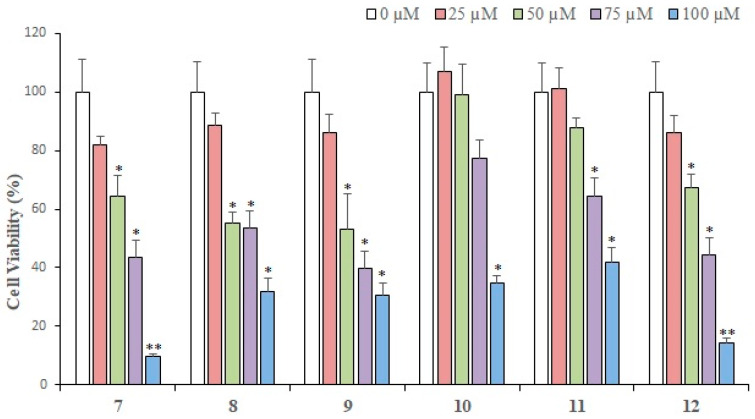
The cytotoxic effects of compounds **7**–**9** (isolated from *L. shawii)* and **10**–**12** (isolated from *A. vera*) on MDA-MB-231 breast cancer cells. All results are expressed as mean ± SEM. * *p* < 0.05 and ** *p* < 0.01, compared to respective untreated controls.

The in vitro cytotoxic activity of each compound isolated from *L. shawii* was examined against breast cancer cells. Results of an MTT assay showed that almost all the compounds exhibited a concentration-dependent growth inhibition of MDA-MB-231 breast cancer cells. At 25 µM (the lowest concentration tested), compound **5** showed maximum (50.53%) loss of cell viability as compared to the rest of the compounds. While **1** exhibited 76% cell viability, **2**, **7**, **9**, **8**, and **3** demonstrated 81–89% cell viability, respectively. Thus, at 25 µM, compound **5** showed a significant cytotoxic effect, while the rest of the compounds did not produce substantial antiproliferative activities. However, at 100 µM (the maximum concentration used to treat cancer cells), compound **1** exhibited maximal inhibition (92.1%) of cell proliferation, followed by compounds **7**, **2**, **3**, **5**, **6**, **4**, **9**, and **8**. Therefore, it is evident that at low concentrations (25 µM and 50 µM), compound **5** is the most active cytotoxic agent [47,48], while at concentrations of 75 µM and above, compound **1** is the most effective cytotoxic compound. Gaweesh et al. (2015) studied the cytotoxic activity of different fractions of *L. shawii* against HepG2, MCF7, and HCT116 cancer cells and reported that the CH_2_Cl_2_ fraction exhibits potent inhibition of the cell growth of MCF7 (breast cancer line) with IC_50_ value of 11 ± 0.195 μg/mL [49]. These active compounds might be potential sources of the anticancer activity of the extracts. Compound **9** was previously isolated by our group [35] which inhibited the cell proliferation in MDA-MB-231 breast cancer cells in a dose-dependent manner. At 25 µM, compound **9** did not inhibit cell growth significantly; however, at concentrations above 50 µM, **9** exhibited over 50% cell growth inhibition in MDA-MB-231 cells. At 100 µM, compound **9** showed approximately 70% loss of cell viability, which is considerable. The results are demonstrated in Figure 2 and Figure 3.

Compounds **10**–**12** were isolated from *Aloe vera* resin and tested for their growth inhibitory potential against MDA-MB-231 cancer cells; **12** demonstrated the highest cytotoxic activities at three different concentrations (Figure 3). At 25 µM, **12** showed ~14.0% of cytotoxic effect, **11** exhibited no cell growth inhibition, while compound **10** depicted a growth-promoting effect on MDA-MB-231 cells. At 50 µM, compounds **10**–**12** exhibited cytotoxic activity in the range of 67–99%, while at 75 µM, **12** showed 44%, while **11** and **10** depicted 64% and 77% cell viability, respectively, indicating that compound **12** is potent at this concentration. At 100 µM, **12** exhibited the highest activity, followed by **10** and **11**. Based on the results, we can say that **1**, **7**, **2**, **3**, **12**, and **5** were the most potential hits. The calculated IC_50_ values revealed that **5**, **1**–**3**, **7**, and **12** are potent cytotoxic compounds with IC_50_ values ranging from 31 to 60 μM, while the IC_50_ values of **8**, **6**, and **9** were 72, 73, and 76 µM, respectively. The IC_50_ values of **1**–**12** are shown in Table 1.

### 2.3. Antioxidant Activity

The antioxidant activity of crude extracts/fractions and isolated compounds was tested using DPPH radical scavengers. The tests were performed at different concentrations to calculate the IC_50_ value. The ethyl acetate, BuOH, and CH_2_Cl_2_ fractions of *L. shawii* showed promising inhibitory potential of 76%, 72%, and 60%, respectively. The IC_50_ values for ethyl acetate, BuOH, and CH_2_Cl_2_ fractions of *L. shawii* were 378 ± 1.50, 650 ± 1.50, and 735 ± 2.00 μg/mL, respectively, indicating that the ethyl acetate fraction is most potent as compared to standard ascorbic acid (53 ± 1.32 μg/mL). The crude MeOH extract of *L. shawii* demonstrated mild activity (50 ± 1.50%), while aqueous and n-hexane fractions were inactive. According to Gaweesh et al. (2015) [49], the EtOAc fraction of aerial parts of *L. shawii* exhibited antioxidant activity with an IC_50_ value of 55.4 ± 3.48 μg/mL. The difference in activity may be due to the composition of the EtOAc fraction using only the stem instead of a mixture (leaves and stem). In the case of *A. vera*, only the ethyl acetate fraction exhibited moderate activity of 51%, while other fractions of this plant did not show promising results. The results are summarized in Table 2.

All the isolated compounds were screened in a DPPH radical scavenging assay in order to test their antioxidant potential. The results demonstrated that compound **4** exhibited potent antioxidant activity with an IC_50_ value of 55 ± 2.00 µM, followed by **14** (IC_50_ = 241 ± 1.50 µM), **6** (IC_50_ = 645 ± 1.50), and **13** (IC_50_ = 762 ± 2.00 µM) as compared to standard ascorbic acid. Compound **4** showed higher activity than **9** (IC_50 =_ 30 µg/mL) reported by our group [35]. The results are tabulated in Table 3. It is a well-accepted notion that the presence and position of –OH groups in a molecule can increase or decrease the antioxidant activity [50]. Among diterpenes, compound **1** showed moderate activity which may be due to the presence of three exocyclic double bonds. Comparing anthraquinones, **6** possesses two –OH groups at the *meta* position of the right hand benzene ring, and one –OH at the C-8 position *meta* to methyl group at C-6. Compounds **5**, **7**, and **8** have similar basic skeletons with the absence of the *meta* –OH group which showed that the meta –OH groups play an important role in the antioxidant activity of anthraquinones. Comparing **4** and **14**, **4** bears two –OH groups at the *meta* position of ring A, two –OH at *ortho* in ring B, one –OH at C-3 in ring C. Compound **14** has two –OH groups at the *meta* position of the benzene ring and one –OH at the C-3 position at cyclohexane. Due to the presence of two additional *ortho* (ring B)–OH groups, compounds showed higher DPPH radical scavenging activity. It is thus suggested that the chelating ability of the *ortho* and *meta* –OH groups in **4** played a greater role in the antioxidation property.

All the isolated compounds were screened in a DPPH radical scavenging assay in order to test their antioxidant potential. The results demonstrated that compound **4** exhibited potent antioxidant activity with an IC_50_ value of 55 ± 2.00 µM, followed by **14** (IC_50_ = 241 ± 1.50 µM), **6** (IC_50_ = 645 ± 1.50), and **13** (IC_50_ = 762 ± 2.00 µM) as compared to standard ascorbic acid. Compound **4** showed higher activity than **9** (IC_50 =_ 30 µg/mL) reported by our group [35]. The results are tabulated in Table 3. It is a well-accepted notion that the presence and position of –OH groups in a molecule can increase or decrease the antioxidant activity [50]. Among diterpenes, compound **1** showed moderate activity which may be due to the presence of three exocyclic double bonds. Comparing anthraquinones, **6** possesses two –OH groups at the *meta* position of the right hand benzene ring, and one –OH at the C-8 position *meta* to methyl group at C-6. Compounds **5**, **7**, and **8** have similar basic skeletons with the absence of the *meta* –OH group which showed that the meta –OH groups play an important role in the antioxidant activity of anthraquinones. Comparing **4** and **14**, **4** bears two –OH groups at the *meta* position of ring A, two –OH at *ortho* in ring B, one –OH at C-3 in ring C. Compound **14** has two –OH groups at the *meta* position of the benzene ring and one –OH at the C-3 position at cyclohexane. Due to the presence of two additional *ortho* (ring B) –OH groups, compounds showed higher DPPH radical scavenging activity. It is thus suggested that the chelating ability of the *ortho* and *meta* –OH groups in **4** played a greater role in the antioxidation property.

### 2.4. Human Intracellular Drug Targets

Compounds **1**–**3**, **5**, **7**, and **12** were identified as the most potentially cytotoxic agents. We utilized cheminformatics techniques [51,52,53,54] to predict the most probable drug targets of these compounds. The kyoto encyclopedia of genes and genome (KEGG) database showed that seven major cancer drug targets are associated with triple-negative breast cancer including epidermal growth factor receptor (EGFR), proto-oncogene tyrosine-protein kinase Kit (c-KIT), insulin-like growth factor receptor 1 (IGFR1), notch receptor 1 (notch 1), phosphatidylinositol-3,4,5-trisphosphate-3-phosphatase (PTEN), phosphatidylinositol-4,5-bisphosphate 3-kinase (PI3K), and cyclin-dependent kinase 4 (CDK4).

The results of the Swiss Target Prediction server are summarized in Table S1 (supporting information) which revealed that compound **1** may target Cyp19A1 with 0.11 probability, which is not an anticancer drug target, while compound **2** depicted 0.09 to <0.05 probabilities for its predicted drug targets. Thus, the results for compound **2** were considered insignificant. Compounds **3** and **5** showed ≥0.10 and ~0.10 probabilities, respectively, for their predicted targets. Among the suggested targets, poly [ADP-ribose] polymerase (PARP)-1 is a potential drug target in TNBC, and carbonic anhydrase II (CA-II), CA-I, CA-XII, CA-IX, and estrogen receptor (ER) α and β are potential anticancer drug targets. However, ER is not expressed in TNBC, thus it was excluded from our study. Compound **7** showed 0.11 probability for its probable targets. Among the suggested targets, CA-II is a target of interest in cancer treatment. Compound **12** showed probabilities in the range of 0.09–0.04 for its suggested targets, in which CA-II was also included, however its probability was lower than 0.05. Based on these results and an extensive literature survey, PARP and CA-II were also included in our docking experiments.

### 2.5. Pharmacophore Modeling

**Figure 4:**
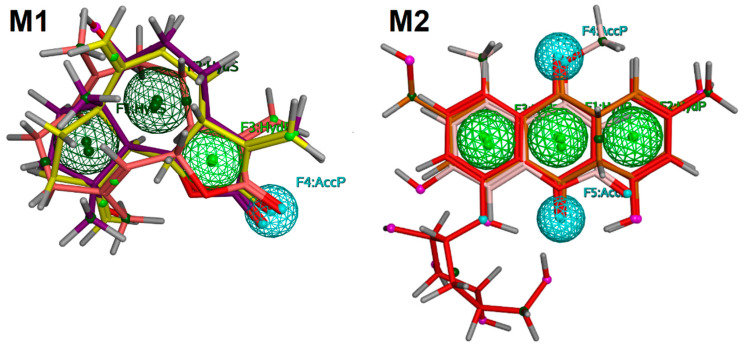
The depiction of pharmacophore models (**M1** and **M2**). The hydrophobic (Hyd) features are shown in green spheres, hydrogen bond acceptor (AccP) features are displayed in cyan spheres. The compounds (shown in stick model) are aligned on their respective pharmacophore models.

The Swiss Target Prediction (STP) server applies 2D-similarity searching to predict the target of the query compound. A number of compounds are present in this server with their actual biological activities and binding mechanism. In STP, 2D structures of the query compounds are matched with the 2D structures of the compounds present in its database and their probabilities are calculated and based on the calculated probabilities, and the target for the query compound is predicted. We also applied pharmacophore modeling to select the drug targets of our compounds with cytotoxic potential. It was hypothesized that the biological targets of those drugs that matched with the pharmacophore model can act as a target of our compounds. Two pharmacophore models, namely, M1 and M2, were generated by using compounds **1**–**3**, **5**, **7**, and **12**. M1 was generated by aligning the 3D structures of compounds **1**–**3**, while M2 was created by aligning the 3D structures of compounds **5**, **7**, and **12**. M1 possessed three hydrophobic (Hyd) and one H-Bond acceptor (AccP) feature while M2 contained three Hyd and two AccP features. The pharmacophore models are displayed in Figure 4.

A set of 62 drug molecules were screened from all the pharmacophore models. M1 retrieved five EGFR inhibitors (afatinib, dacomitinib, nazartinib, neratinib, pelitinib), three PI3K inhibitors (apitolisib, leniolisib, samotolisib), and one CDK4 inhibitor (*Omacetaxine mepesuccinate*). M2 identified six inhibitors of c-KIT (dovitinib lactate, midostaurin, ripretinib, semaxanib, toceranib, and sunitinib), one Notch 1 (crenigacestat), and two PI3K (dactolisib, samotolisib) inhibitors. This pharmacophore-based searching reflects that compounds **1**–**3** may target EGFR, PI3K, and CDK4 while compounds **5**, **7**, and **12** may bind with c-KIT, Notch 1, and PI3K. Subsequently, compounds **1**–**3**, **5**, **7**, and **12** were subjected to structure-based inverse docking to further confirm the results of pharmacophore modeling.

### 2.6. Molecular Docking Studies

**Figure 5:**
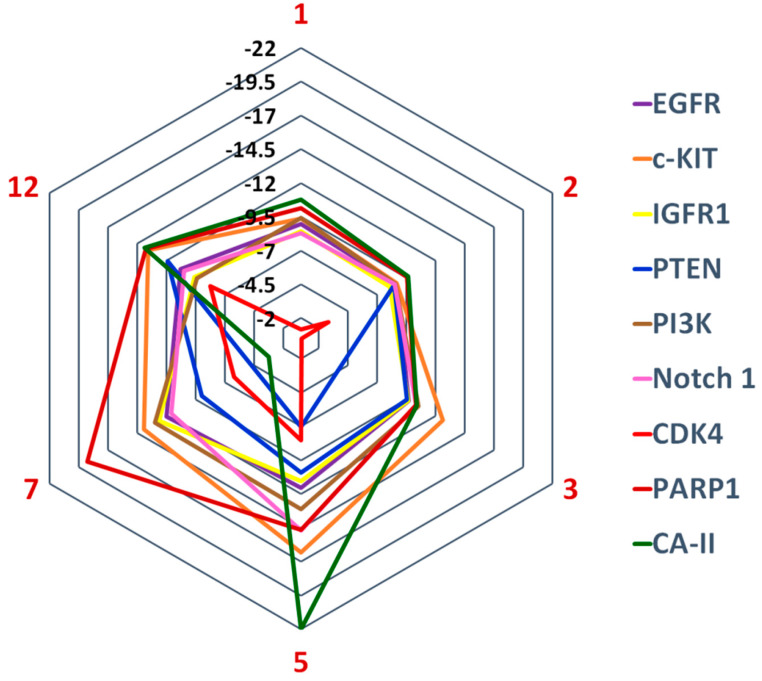
Graphical presentation of predicted cellular targets of Compounds **1**–**3**, **5**, **7**, and **12** based on docking scores.

Compounds **1**–**3**, **5**, **7**, and **12** were potential hits in our cell-based anticancer assay. Structure-based inverse docking analysis was carried out to predict their binding mechanism. Compounds were docked at the active site or ligand binding sites of the selected drug targets. The docking scores of the compounds suggests that CA-II is the most potential target for compounds **1** (−10.75), **2** (−9.64), **5** (−21.98), and **12** (−13.88), while c-KIT is the best target for compound **3** (−12.62). After CA-II, PARP-1 is an excellent candidate for **1**, **2**, **7**, and **12**, while PI3K and c-KIT are probable drug targets for compounds **3** and **5**, respectively. Moreover, c-KIT is a good drug target for compounds **2**, **7**, and **12**, while PARP1 is the fourth good target for compounds **3** and **5**. PI3K was identified as the second most probable drug target for compound **3**, while moderate for **1**, **7**, **2**, and **5**, and least for **12**. The docking score suggests that the receptors EGFR, Notch 1, and IGFR have variable potency with these compounds, however, binding interactions suggest that compound **7** possesses good binding interactions with Notch 1, while compound **5** has higher binding interactions with EGFR. As per the docking scores, PTEN was the least favorable target for compounds **1**–**3**, **5**, and **7**, while CDK4 was ranked as the worst target for all the compounds (Figure 5). The docking results are presented in Table S2.

**Figure 6:**
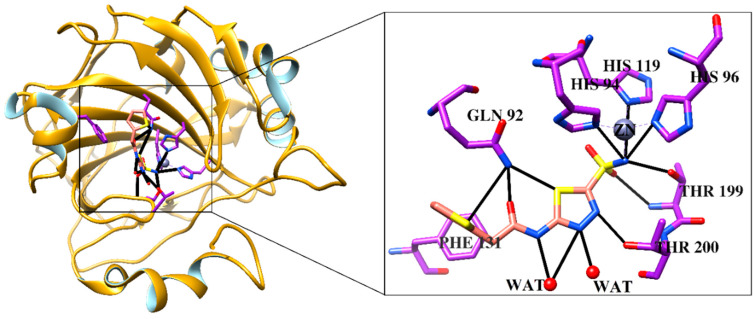
The 3D structural topology of human carbonic anhydrase II is displayed in ribbon form complex with known inhibitor. The active site residues are shown in purple stick model, ligand is depicted in coral sticks, hydrogen bonds are displayed as black lines.

**Figure 7:**
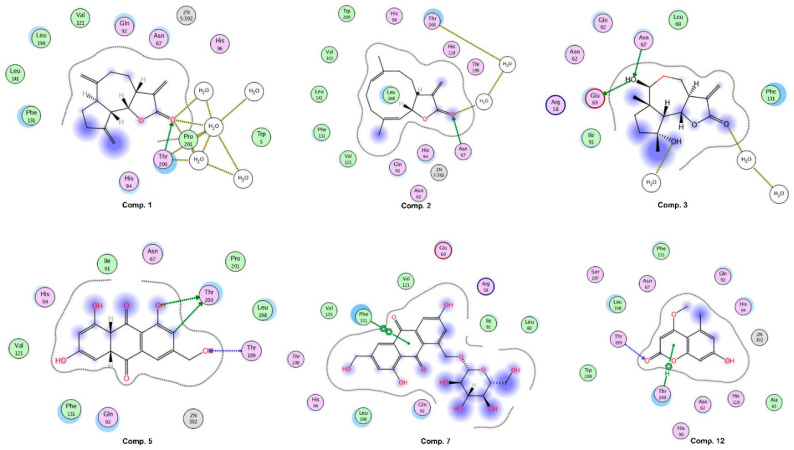
The binding interactions of compounds **1**–**3**, **5**, **7**, and **12** at the active site of human carbonic anhydrase II. Side chain donors/acceptors are depicted in green arrows, backbone donors/acceptors are shown in blue arrows, solvent contact is presented in yellow dotted lines.

CA-II was exposed as the best target for compounds **1**, **2**, **5**, and **7**. Compound **1** binds with the docking score −10.75 and interacts with three water molecules and the side chain of Thr200 by H-bonding. Compound **2** interacts with the side chain of Asn67 and one water molecule via H-bonding. The –OH groups of compound **3** accept and donate H-bonds to the side chains of Asn67 and Glu69, respectively. Moreover, two water molecules provide H-bonding to the compound. The docked view of compound **5** depicts that its –OH groups donate H-bonds to the side chain of Thr200 and accepts H-bonds from the amino group of Thr199, while its ring mediates hydrophobic interactions with the side chain of Leu198. The docking score (−21.98) of **5** suggests that this compound possesses the highest binding potential for CA-II in silico. The side chain of Phe131 provides π-π interactions to the ring of compound **7**. The docking score of **7** (−3.26) and its binding interactions suggest that this compound is the least active as compared to the rest of the compounds. The carbonyl moiety of compound **12** mediates bidentate interactions with the side/main chain amino groups of Thr199. Moreover, hydrophobic interaction was observed between the ring of the compound and the side chain of Thr199. The docking score of **12** (−13.88) is less than the docking score of compound **5**, while higher than the docking scores of compounds **1**–**3** and **7**. Some of the docked conformations of **12** showed that the compound may bind with the Zn atom present in the active site. The reference known drug, acetazolamide, interacts with ZN via metal–ligand interaction, and its carbonyl group also accepts H-bonds from the side chain of Thr199. The sulphate oxygen and ring nitrogen also mediate several water-mediated interactions. The active site of human CA-II is presented in Figure 6. The ligand (1GO), an acetazolamide derivative, complexed in the X-ray structure of CA-II, is also displayed, which interacts with ZN, His94, His96, His119, Gln92, Phe131, Thr199, Thr200, and two water molecules. Our compounds, except **12**, do not bind with the zinc (ZN) present in the active site; however, they interact within the vicinity. The binding interactions of compounds are shown in Figure 7. The docking results are tabulated in supporting information Table S2.

After CA-II, PARP-1 was the second most probable target for compounds **1**, **2**, **7**, and **12**, while fourth for compounds **3** and **5**. Among all the compounds, **7** (−18.74), **5** (−14.64), and **12** (−13.74) demonstrated higher binding scores than the niraparib (standard drug or positive control for PARP1, −13.18), while compounds **3** (−10.32), **1** (−10.14), **2** (−9.56) displayed lower scores than **7**, **5**, **12**, and niraparib. At the active site of PARP-1, Glu988 and Ser904 provided H-bonds to compound **5**, while Glu988 and a water molecule stabilized compound **7** via H-bonding. At the active site of c-KIT, compound **5** displayed the highest binding potential with docking score −16.31 as compared to the rest of the compounds, while compounds **7** (−13.91) and **12** (−13.56) displayed comparable docking scores. The binding modes also demonstrated the reason for higher binding scores. Compounds **5** and **7** had higher numbers of H-bonding and hydrophobic interactions as compared to **1**–**3** and **12**. Moreover, compounds **5**, **7**, **12**, and **3** depicted higher docking scores than the reference compound sunitinib, which is a drug complexed in the X-ray crystal structure of c-KIT (used in docking studies). Thr670, CYS673, LEU595, VAL603, and a water molecule (HOH289) played important roles in the stabilization of compounds **5**, **7**, and **12** in the active site. These residues provided hydrogen bonding and π–H interactions to the compounds.

Compounds **7** and **5** displayed higher binding scores than the positive control of EGFR (afatinib) and IGFR (ibutamoren mesylate). While compounds **5**, **7**, **12**, and **3** scored better than crenigacestat (inhibitor of Notch 1), compounds **12**, **5**, and **7** were better than inhibitors of CDK4 (omacetaxine mepesuccinate), CA-II (acetazolamide) and PARP1 (niraparib). The docking results are tabulated in Table S1.

### 2.7. Inhibition of Carbonic Anhydrase II (CA-II) by Compounds **1**, **2**, **5**, **7**, and **12**

Based on docking results, compounds **1**, **2**, **5**, **7**, and **12** were tested for their potential against CA-II in vitro. The quantity of compound **3** was not sufficient, thus it was not tested. The in vitro testing results showed that compound **5** is the most active compound with IC_50_ values of 14.4 µM, followed by compounds **12** and **2**. Both the compounds (**12** and **2**) also showed very good activity in the range of 23 and 24 µM, respectively. The docking score also indicated that compounds **5** and **12** are the most active compounds. In docking, compounds **1** and **3** showed good binding potential after **5** and **12**, however, in vitro results demonstrated that compound **1** is inactive. Compound **7** showed the least binding potential in silico and was found to be inactive against CA-II in vitro. The results are summarized in Table 4. The structure–activity relationship of the compounds is discussed in docking results. The experimental findings correlate well with the docking results.

## 3. Materials and Methods

This study consisted of extraction and isolation of bioactive compounds from *L. shawii* and *A. vera* and determination of their cytotoxic and antioxidant potential. The cytotoxic assay was conducted on TNBC cell lines, followed by in silico molecular targeting of active compounds. Pharmacophore modeling and molecular docking approaches were used in computational analysis. The computational results were validated by in vitro testing of active compounds against the most suitable predicted targets.

### 3.1. General Instrumentation

NMR spectra were recorded on an NMR spectrometer (BRUKER, Zürich, Switzerland) operating at 600 MHz with cryoprobe prodigy (150 MHz for ^13^C; chemical shift (δ) = ppm; coupling constants (*J*) = Hz). Infrared (IR) spectra were recorded on an ATR-Tensor 37 spectrophotometer, Bruker (Ettlingen, Baden-Württemberg, Germany). ESI-MS spectra were recorded on a mass spectrometer (Waters Quattro Premier XE, Waters, Milford, MA, USA). For thin-layer chromatography (TLC, silica gel 60F-254, Merck, Darmstadt, Hesse, Germany), precoated aluminum sheets were used. TLC plates were visualized under UV light at 254 and 366 nm and mostly sprayed with the ceric sulfate (Ce(SO_4_)_2_) reagent followed by heating with heating gun.

### 3.2. Plant Material and Identification

The whole plant materials of *L. shawii* and *A. vera* resin were purchased from market (Souq, Nizwa) and identified by the plant taxonomists Saif Al-Hatmi (Oman Botanical Garden, Muscat, Oman (OBGM)) and Syed Abdullah Gillani (Department of Biological Sciences and Chemistry (DBSC), University of Nizwa, Oman), respectively. Voucher specimens of *A. vera* (No. AFS-08/2016) and *L. shawii* (No: BSHR-05/2015) were deposited in the herbarium of OBGM and DBSC, respectively.

### 3.3. Extraction, Fractionation, and Isolation of Bioactive Compounds

The air-dried stem powder material of *L. shawii* was extracted with methanol for two weeks. The resulting methanol extract was suspended in distilled water (H_2_O) and successively partitioned into *n*-hexane, dichloromethane (CH_2_Cl_2_), ethyl acetate (EtOAc), n-butanol (*n*-BuOH), and aqueous (H_2_O) fractions. The *n*-hexane fraction was subjected to silica gel column chromatography (70–230 mesh; Merck) and produced two compounds, **1** and **2** [22]. Similarly, the EtOAc fraction was subjected to CC and eluted with an increasing polarity, viz., *n*-hexane–EtOAc, EtOAc, EtOAc–MeOH, and pure MeOH, to isolate seven compounds, **3**–**9** [35,55].

Similarly, the shade-dried powdered resin of *A. vera* was exhaustively extracted with MeOH (2 L) at room temperature (3 × 15 days). Evaporation of the MeOH in vacuo at 45 °C yielded a crude methanol extract, which after suspension in water was successively fractionated into *n*-hexane, CH_2_Cl_2_, EtOAc, and *n*-BuOH [36]. After taking TLC, CH_2_Cl_2_ and EtOAc fractions were combined. The combined material was subjected to CC and eluted with *n*-hexane, *n*-hexane–EtOAc, EtOAc, MeOH/EtOAc, and finally washed with 20% MeOH/EtOAc with 10% increments in polarity to afford several fractions which were subsequently subjected to further repeated CC with different concentrations of *n*-hexane-EtOAc-MeOH as eluent to obtain 11 compounds, **10**–**20** [36].

### 3.4. Assay Protocol for Cytotoxic Activity

Cytotoxic activity was performed according to the previously described methods [56,57]. Breast cancer cell lines MDA-MB-231 were maintained in Dulbecco’s modified eagle medium (DMEM, Invitrogen, Carlsbad, CA, USA). The media was supplemented with 10% fetal bovine serum (FBS) and 1% antimycotic antibiotic (Invitrogen, Carlsbad, CA, USA). Cells were cultured in a 5% CO_2_-humidified atmosphere at 37 °C. Stock solution (5 mg/mL) of 3-(4,5-dimethylthiazol-2-yl)-2,5-diphenyltetrazolium bromide (MTT, Merck, Darmstadt, Hesse, Germany) was prepared in phosphate-buffered saline (PBS).

Cells were seeded at a density of 1 × 10^4^ cells per well in 96-well microtiter culture plates. After overnight incubation, normal growth medium was removed and replaced with either fresh medium (untreated control) or different concentrations of respective compounds in growth medium diluted from a 2 mM stock. After 24 h of incubation, MTT solution was added to each well (0.1 mg/mL in DMEM) and incubated further for 4 h at 37 °C. Upon termination, the supernatant was aspirated and the MTT formazan, formed by metabolically viable cells, was dissolved in a solubilization solution containing DMSO (100 սL) by mixing for 5 min on a gyratory shaker. The absorbance was measured at 540 nm (reference wavelength 690 nm) on an Ultra Multifunctional Microplate Reader (Bio-Rad, Hercules, California, USA). Absorbance of control (without treatment) was considered as 100% cell survival. Each treatment had three replicate wells [56]. The IC_50_ values of the active compounds were calculated using nonlinear regression through GraphPad Prism 4 software (La Jolla, CA, USA).

### 3.5. Assay Protocol for DPPH Radical Scavenging Activity

Free radical scavenging activity of the test fractions/compounds was determined by measuring the change in absorbance of DPPH (l, l-Diphenyl-2-picrylhydrazyl radical, Sigma-Aldrich, St Louis, MO, USA) at 515 nm by the microplate reader (SpectraMax M2, Molecular Devices, CA, USA) as previously described [57]. Ascorbic acid (Sigma-Aldrich, St Louis, MO, USA) was used as a standard with 90% inhibition and IC_50_ value of 53 µg/mL. The IC_50_ values of the tested compounds were calculated using nonlinear regression through GraphPad Prism 4 software (La Jolla, CA, USA).

### 3.6. In Silico Target Fishing

The coordinates of six compounds (**1**–**3**, **5**, **7**, and **12**) were generated by ChemDraw Ultra 10 (PerkinElmer Inc.) [58] and converted into three-dimensional (3D) form using molecular operating environment (MOE version 2013.08) [59]. The SMILE formats of the compounds (**1**–**3**, **5**, **7**, and **12**) were uploaded on the Swiss Target Prediction webserver (http://www.swisstargetprediction.ch/) to predict the intracellular drug targets of these compounds in humans. In addition, the Kyoto Encyclopedia of Genes and Genomes (KEGG) database (https://www.genome.jp/kegg/) was used to identify the probable targets of our active compounds in human triple-negative breast cancer (TNBC). The KEGG pathway (HSA05224 and H00031) showed that epidermal growth factor receptor (EGFR), proto-oncogene tyrosine-protein kinase Kit (c-KIT), insulin-like growth factor 1 receptor (IGF1R), and Notch receptor 1 (Notch 1) are particularly overexpressed in TNBC. Moreover, phosphatidylinositol-3,4,5-trisphosphate-3-phosphatase and dual-specificity protein phosphatase (PTEN), phosphatidylinositol-4,5-bisphosphate 3-kinase (PI3K), and G1/S-specific cyclin-D1 (CCND1 or CDK4) are found to be mutated in TNBC. Thus these targets were scrutinized in the molecular docking protocol.

### 3.7. Selection of Known Drugs

By extensive literature survey, 62 known anticancer drugs were selected and their 3D structures were taken from the PubChem database (https://pubchem.ncbi.nlm.nih.gov/), which was used as a library in pharmacophore searching. Moreover, during docking, those drugs were considered as positive control or reference compounds. The docking results of compounds **1**–**3**, **5**, **7**, and **12** were compared with the docking scores of these drugs. The selected drugs for each target are tabulated in Table 5.

### 3.8. Pharmacophore Modeling

Pharmacophore modeling was performed by MOE-Pharmacophore editor using PPCH-ALL annotation scheme. PPCH-ALL has H-bond Donor (HBD), H-bond Acceptor (HBA) and their Projections, π vs. non-π H-bond Donor/Acceptor, General π vs. non-π Distinctions, Metal Ligator, Metal Ligator Projection, Cation, Anion, and Hydrophobe (HYD) terms. The pharmacophore models were generated by using 3D structures of active compounds **1**–**3**, **5**, **7**, and **12**. Compounds **1**–**3** were aligned and model 1 was generated, while model 2 was generated by aligning the compounds **5**, **7**, and **12**. The common pharmacophoric features were selected in the aligned compounds.

### 3.9. Molecular Docking

The X-ray crystal structures of the target proteins including EGFR, c-KIT, IGF1R, Notch 1, PTEN, PI3K, CDK4, PARP1, and CA-II were retrieved from Research Collaboratory for Structural Bioinformatics Protein Data Bank (RCSB-PDB) (https://www.rcsb.org/). The data are summarized in Table 6. For docking, protein files were prepared by addition of protons, partial charges, and the removal of cocrystallized ligands and heteroatoms. The role of water molecules was deduced by visualizing the protein–ligand interactions. Only water molecules within the vicinity of 3.0 Å of active ligands were retained in the file during docking, otherwise they were removed from the protein structure. The structures of the compounds and selected drugs were energy minimized using MMFF94x forcefield and gradient: 0.05. During energy minimization, hydrogens were added, and partial charges were applied.

Molecular docking was performed by MOE using Triangle Matcher placement method, Rescoring1: London dG, Refinement: Forcefield, and Rescoring2: Affinity dG. As a default parameter, 30 docked conformations were selected to be saved for each compound after docking. After docking, we applied conformation sampling method to select the best docked orientation of compound. For this purpose, each conformation of all the docked compounds were visualized and based on the protein–ligand interactions, docking score, and rank, best conformation was selected for analysis. The images in 2D were captured through MOE ligand binding interaction while 3D images were taken by Chimera [60].

### 3.10. Carbonic Anhydrase II Inhibition

The total reaction volume of 200 µL included 20 µL of test compounds prepared in DMSO, followed by the addition of 140 µL of the HEPES–tris buffer, 20 µL of purified bovine erythrocyte CA-II (0.1 mg/mL) prepared in buffer, and 20 µL of a solution of 4-nitrophenyl acetate [70]. A 20 µL amount of test compound was incubated with the enzyme (EC 4.2.1.1, Sigma-Aldrich, St. Louis, MO, USA) for 15 min in a 96-well flat-bottom plate. The rate of product formation was monitored with the addition of 20 µL of 4-NPA as substrate, prepared in methanol at the final concentration of 0.7 mM, at 25 °C for 30 min with regular intervals of 1 min, by using microplate readers (Bio-Rad, Molecular Devices, CA, USA). HEPES-tris was used as a buffer for the reaction at the final concentration of 20 mM at pH 7.4. The percent inhibition was calculated by using the following formula:

% Inhibition = 100 − (OD test well/OD control) × 100

### 3.11. Statistical Analysis for Cytotoxic Activities

Results are expressed as mean ± SEM of at least three independent observations. Student’s t-test was used to statistically examine significant differences. Analysis of variance was performed using ANOVA. *p*-Values < 0.05 were considered statistically significant.

## 4. Conclusions

Twenty compounds (isolated from *L. shawii* and *A. vera*) were scrutinized for their anticancer potential in triple-negative breast cancer cell lines (MDA-MB-231). Among the tested compounds, compounds **5**, **1**–**3**, **7**, and **12** were retrieved as most potential hits. We used extensive in silico target fishing techniques to predict their biological targets in the human genome. For this purpose, 2D-cheminformatic tools and 3D-pharmacophore modeling were used that suggested that carbonic anhydrase II (CA-II), poly [ADP-ribose] polymerase (PARP)-1, and proto-oncogene tyrosine-protein kinase Kit (c-KIT) can be possible targets for the active hits. The in silico findings were validated by in vitro testing of compounds on CA-II, which showed that **5**, **12**, and **2** are excellent inhibitors of CA-II. Moreover, antioxidant activities of compounds were examined, which demonstrated that compound **4** possesses the highest antioxidant potential. These results indicated that constituents of *L. shawii* and *A. vera* are promising drug candidates for triple-negative breast cancer and should be investigated in detail.

## Figures and Tables

**Table 1:** The calculated IC_50_ values of compounds **1**–**12** in MDA-MB-231 breast cancer cell lines.

Compounds	IC_50_ (Mean ± SEM) (μM)
**1**	**35.36 ± 2.56**
**2**	**42.08 ± 2.98**
**3**	**49.39 ± 3.73**
**4**	101.4 ± 7.09
**5**	**31.36 ± 2.44**
**6**	73.47 ± 5.89
**7**	**57.32 ± 4.14**
**8**	72.21 ± 6.29
**9**	76.9 ± 7.04
**10**	142.8 ± 12.66
**11**	140.4 ± 13.1
**12**	**60.09 ± 4.82**
Doxorubicin (+ve control)	3.31 ± 0.19

Highly active compounds are highlighted in bold.

**Table 2:** Antioxidant activity of different fractions of *L. shawii* and *A. vera* resin.

Antioxidant % Inhibition (IC_50_ ± SEM)			
Code	*L. shawii*	Code	*A. vera*
BF	72 (650 ± 1.50)	MF	NA
MF	50	EF	51
WF	NA	DF	42
HF	NA	BF	32
EF	76 (378 ± 1.50)	WF	35
DF	60 (735 ± 2.00)	HF	NA
Ascorbic acid	90 (53 ± 1.32)		

IC_50_ = μg/mL; concentration = 1 mg/mL; NA = not active; BF = n-butanol, MF = methanol, WF = aqueous, HF = hexane, EF = ethyl acetate, DF = dichloromethane.

**Table 3:** Antioxidant activity of the active compounds.

Numbering	% Inhibition (1 mM)	IC_50_ ± SEM (μM)
**4**	78	55 ± 2.0
**6**	71	645 ± 1.5
**13**	73	762 ± 2.0
**14**	80	241 ± 1.5

SEM = Standard Error Mean.

**Table 4:** The anti-CA-II activities of compounds **1**, **2**, **5**, **7**, and **12**.

Compounds	Docking Score	% Inhibition	IC_50_ (µM) ± (SEM)
**1**	−10.75	33	NA
**2**	−9.64	84.7	24.4
**3**	−10.39	NT	NT
**5**	−21.98	86.3	14.4 ± 1.14
**7**	−3.26	37.5	NA
**12**	−13.88	91.2	23.3 ± 1.63

SEM = Standard Error Mean; NA = Not Active; NT = Not Tested.

**Table 5:** Known drugs against the selected drug targets.

Target	Drugs
EGFR	Afatinib, Canertinib dihydrochloride, Dacomitinib, Erlotinib, Gefitinib, Icotinib, Lapatinib, Lifirafenib, Masoprocol, Mavelertinib, Naquotinib, Nazartinib, Neratinib, Olmutinib, Osimertinib, Pelitinib, Rociletinib, Vandetanib, Varlitinib
c-KIT	Amuvatinib, Ancestim, Avapritinib, Cabozantinib, Dasatinib, Dovitinib lactate, Imatinib, Masitinib, Midostaurin, Motesanib, Nilotinib, Pazopanib, Regorafenib, Ripretinib, Semaxanib, Sorafenib, Sunitinib, Tandutinib, Toceranib, Vatalanib
IGFR1	Ibutamoren mesylate, Linsitinib, Mecasermin, Mecasermin rinfabate, Toremifene
Notch 1	Crenigacestat
PI3K	Apitolisib, Bimiralisib, Buparlisib, Dactolisib, Gedatolisib, Leniolisib, Omipalisib, Pictilisib, Samotolisib
CDK2	Omacetaxine mepesuccinate
PARP	Olaparib, Niraparib, Rucaparib, Talazoparib, Veliparib
CA-II	Acetazolamide

**Table 6:** The selected PDB structures for each anticancer drug target.

S #	Target	PDB ID	Ligand ID	Resolution (Å)	References
**1**	EGFR	2G5J	0WN (Afatinib)	2.8	[61]
**2**	c- Kit	3G0E	B49 (Sunitinib)	1.6	[62]
**3**	IGFR1	3F5P	741 (3-Cyanoquinoline)	2.9	[63]
**4**	Notch 1	3L95	Antibody FAB fragment	2.19	[64]
**5**	PTEN	5BZX	VO4 (bisperoxovanadium complex)	2.5	[65]
**6**	PI3K	5ITD	6CY (5-{4-[3-(4-acetylpiperazine-1-carbonyl)phenyl]quinazolin-6-yl}-2-methoxypyridine-3-carbonitrile)	3.02	[66]
**7**	CDK4	2W9Z	Cyclin D	2.45	[67]
**8**	PARP1	4R6E	3JD (Niraparib)	2.2	[68]
**9**	CA-II	4IWZ	1GO (acetazolamide derivative)	1.598	[69]

## Supplementary Materials

The following are available online at https://www.mdpi.com/1424-8247/13/5/94/s1, Table S1: Human intra-cellular targets—swiss target prediction results. Table S2: Molecular docking scores and binding interactions of compounds **1**–**3**, **5**, **7**, and **12** on the selected drug targets. Figure S1: ^1^H, ^13^C-NMR and ESI-MS spectra of the known compounds (**1**–**20**).
