# Supplementary material for: Antiproliferative and Carbonic Anhydrase II Inhibitory Potential of Chemical Constituents from Lycium shawii and Aloe vera: Evidence from In Silico Target Fishing and In Vitro Testing
Source: Pharmaceuticals (Basel). 2020 May 13;13(5):94. doi: 10.3390/ph13050094 (PMC7281707; doi:10.3390/ph13050094)
Supplement: Supplementary file 1 [file pharmaceuticals-13-00094-s001.zip › pharmaceuticals-774902-supplementary.doc]

**Supporting Information**

Antiproliferative and Carbonic Anhydrase II Inhibitory Potential of Chemical Constituents from *Lycium shawii* and *Aloe vera*: Evidence from In Silico Target Fishing and In Vitro Testing

Najeeb Ur Rehman ^1,†^, Sobia Ahsan Halim ^1,†^, Majid Khan ^1,2^, Hidayat Hussain ^1,3^, Husain Yar Khan^1^, Ajmal Khan ^1^, Ghulam Abbas ^4^, Kashif Rafiq ^1,5^ and Ahmed Al-Harrasi ^1,^*

^1^	Natural & Medical Sciences Research Center, University of Nizwa, P.O Box 33, 616, Birkat Al Mauz, Nizwa, Postal Code 616, Sultanate of Oman; najeeb@unizwa.edu.om (N.U.R.); sobia_halim@unizwa.edu.om (S.A.H.); majid.khan@unizwa.edu.om (M.K.); hussainchem3@gmail.com (H.H.); husainyar@gmail.com (H.Y.K.); ajmalkhan@unizwa.edu.om (A.K); kashifrafiq609@gmail.com (K.R.)

^2^	HEJ Research Institute of Chemistry, University of Karachi, Karachi-75270, Pakistan

^3^	Department of Bioorganic Chemistry, Leibniz Institute of Plant Biochemistry, 06120 Halle, Germany

^4^	Department of Biological Sciences and Chemistry, University of Nizwa, P.O Box 33, 616, Birkat Al Mauz, Nizwa, Sultanate of Oman; abbashej@unizwa.edu.om

^5^	Department of Chemistry, Abdul Wali Khan University Mardan, Mardan 23200, Pakistan

Correspondence: aharrasi@unizwa.edu.om; Tel**:** +968-25446328; Fax: +968-2544-6612

^†^	Both the authors contributed equally.

Received: 31 March 2020; Accepted: 4 May 2020; Published: date

**Table S1. Human intra-cellular targets – swiss target prediction results**

| **Compounds** | Targets | **Probability** |
| --- | --- | --- |
| **1** | cytochrome P450 (Cyp450) 19A1 | 0.11 |
|  | cyclooxygenase-2 (COX-2), thrombin and trypsin I | 0.06 |
|  | chymotrypsin C, 11-β-hydroxysteroid dehydrogenase 1, potassium channel subfamily K member 2, Epoxide hydrolase 1, CypP450 11B1 and B2, DNA polymerase β, poly [ADP-ribose] polymerase-1, dopamine D4 receptor, thromboxane-A synthase, Leucine-rich repeat serine/threonine-protein kinase 2, cathepsin K, tyrosine-protein kinase receptor FLT3, estradiol 17β-dehydrogenase 3, alcohol dehydrogenase α, MAP kinase p38α, glycogen synthase kinase 3β, thymidylate synthase, myeloperoxidase, androgen Receptor, Cyp450 17A1, Exportin-1, adrenergic receptor α-2, steroid 5α-reductase 1, PI3-kinase p110δ/p85α, Cyp450 2A6, and COX-1 | <0.05 |
| **2** | Cyp19A1 | 0.09 |
|  | Androgen receptor | 0.06 |
|  | protein farnesyltransferase, progesterone receptor, Interleukin-1β, tubulin tyrosine ligase, HMG-CoA reductase, phosphodiesterase 4D, protein-tyrosine phosphatase 1B, glycogen synthase kinase-3β, Cyp450 11B1 and B2, thromboxane-A synthase, thymidylate synthase, tyrosine-protein kinase receptor FLT3, subtilisin/kexin type 7, potassium-transporting ATPase α2, myeloperoxidase, poly [ADP-ribose] polymerase-1, DNA polymerase α/β, COX-2, alcohol dehydrogenase α, cathepsin K, epoxide hydrolase 1, potassium channel subfamily K member 2, protein kinase C α, dual specificity phosphatase Cdc25C, programmed cell death protein 4, DNA-dependent protein kinase, 11β-hydroxysteroid dehydrogenase 1, carbonic anhydrase II and III (CA-II and CA-III), leucine-rich repeat serine/threonine-protein kinase 2, and alcohol dehydrogenase γ | <0.05 |
| **3** | COX-2 | 0.16 |
|  | Cyp19A1 | 0.13 |
|  | DNA polymerase β, 11β-hydroxysteroid dehydrogenase 1, bromodomain-containing protein 4, bromodomain-containing protein 2, protein kinase Cα, FK506-binding protein 1A, CDC7/DBF4 (Cell division cycle 7-related protein kinase/Activator of S phase kinase), thrombin, trypsin I, chymotrypsin C, tyrosine-protein kinase JAK1, COX-1, dopamine D2 receptor, androgen receptor, glucocorticoid receptor, JAK3, JAK2, Tyrosine-protein kinase TYK2, inducible Nitric oxide synthase, telomerase reverse transcriptase, prostaglandin E synthase, voltage-gated potassium channel subunit Kv1.5, P-glycoprotein 1, DNA polymerase α, transient receptor potential cation channel subfamily M member 8, CREB-binding protein/p53, leucine-rich repeat serine/threonine-protein kinase 2, adrenergic receptor β, β1 adrenergic receptor (AR), β3-AR, Cyp450 11B1, Cyp450 11B2, DNA-dependent protein kinase, macrophage migration inhibitory factor, cyclin-dependent kinase 5/CDK5 activator 1, poly [ADP-ribose] polymerase-1, PI3-kinase p110δ, PI3-kinase p110β, programmed cell death protein 4, myeloperoxidase, matrix metalloproteinase (MMP) 9, MMP-1 and MMP-8 | ≤ 0.11 |
| **5** | MMP-3, MMP-1, ADAM17, purine nucleoside phosphorylase, CA-II, CA-I, CA-XII. CA-IX, MMP-7, MMP-12, cytidine deaminase, MMP-8, thymidine phosphorylase, adenosine deaminase, estrogen receptor α and β | ~0.10 |
| **7** | Sodium/glucose cotransporter 1, CA-II, CA-I, CA-XII, equilibrative nucleoside transporter 1, MMP-13, MMP-1, MMP-7, MMP-12, MMP-8, β-glucocerebrosidase, adenosine A2b receptor, EGFR-1, low affinity sodium-glucose cotransporter, CA-XIV, adenosine kinase, DNA topoisomerase I,Type-1 angiotensin II receptor, sodium/nucleoside cotransporter 2, Voltage-gated potassium channel subunit Kv1.3, tyrosinase, CDK-1, transforming protein p21/H-Ras-1, HSP-71, MMP-3, ADAM17, bifunctional protein NCOAT 78 kDa glucose-regulated protein, hexokinase type II and I, HSP 90α, leukocyte elastase, α-L-fucosidase I, neprilysin, Inosine-5'-monophosphate dehydrogenase (IMPDH) 1, IMPDH-2, c-Jun kinase 3, Inhibitor of NF-κβ kinase β-subunit, galectin-4, adenosine deaminase, transthyretin, galectin-3, galectin-8, tyrosine-protein kinase ABL, ephrin type-A receptor 2, MAPKKK9, tyrosine-protein kinase BTK, tyrosine-protein kinase LCK, CDK2/cyclin A, tyrosyl-tRNA synthetase, dual specificity phosphatase Cdc25A, glyceraldehyde-3-phosphate dehydrogenase, serine/threonine-protein kinase Chk1, methionyl-tRNA synthetase, histone deacetylase 1, Insulin-like growth factor binding protein 3, purine nucleoside phosphorylase, dual specificity mitogen-activated protein kinase kinase 1, and thymidine phosphorylase. | 0.11 |
| **12** | Estradiol 17-βDH3, CA-XII, | 0.09 |
|  | CA-IX, CA-VII | 0.08 |
|  | Estrogen receptor (ER) α and β | 0.06 |
|  | CA-III, CA-I, CA-XIV, and Monoamine oxidase (MAO) B | 0.05 |
|  | Myoglobin, MAO-A, CA-II, CA-XIII, fatty acid desaturase 1, γ-amino-N-butyrate transaminase, Carboxylesterase 2, dual specificity protein kinase CLK1, dual specificity tyrosine-phosphorylation-regulated kinase 1B, serine/threonine-protein kinase/endoribonuclease IRE1, acetylcholinesterase, plectin, casein kinase (CK) Iα, CK Iδ, serine/threonine-protein kinase Aurora-B, CA-VA, GPCR kinase 6, Aldehyde dehydrogenase, arylamine N-acetyltransferase 1, catechol O-methyltransferase, alkaline phosphatase, tissue-nonspecific isozyme, CA-IV, Calcium-activated potassium channel subunit α1, Cyp450 19A1, CA-VI, Interleukin-8RA, purine nucleoside phosphorylase, alkaline phosphatase placental-like, phospholipase A-2-activating protein, GPCR55, androgen receptor, succinate semialdehyde dehydrogenase, EGFR1, CDK-2, CDK-4, ribonuclease H1, phenylethanolamine N-methyltransferase, histone deacetylase 2, histone deacetylase 8, voltage-gated potassium channel subunit Kv1.3, Cyp450 1A2 | 0.04 |

**Table S2.** Molecular docking scores and binding interactions of compounds **1**-**3**, **5**, **7**, and **12** on the selected drug targets

| **Targets** | **Compounds** | **Score** | **Binding Interactions (Å)** | | | |
| --- | --- | --- | --- | --- | --- | --- |
|  |  |  | **Ligand** | **Receptor** | **Interactions** | **Distance** |
| **EGFR** | **1** | -8.97 | O18 | NZ-LYS745 | HBA | 2.82 |
|  | **2** | -8.63 | O17 | NZ-LYS745 | HBA | 1.68 |
|  | **3** | -9.70 | O12  O17 | NZ-LYS745  N-MET793 | HBA  HBA | 2.77  3.09 |
|  | **5** | -11.55 | O15  O19  O17  O21 | O-MET793  OG1-THR790  N-MET793  N-ASP855 | HBA  HBD  HBA  HBA | 2.06  1.84  2.47  1.30 |
|  | **7** | -12.01 | O28  O17 | O-MET793  NZ-LYS745 | HBD  HBA | 1.55  1.99 |
|  | **12** | -10.79 | O13  O | OE2-GLU762  N-MET793 | HBD  HBA | 2.07  2.65 |
|  | **Afatinib** | -11.06 | N7 | N-MET793 | HBA | 2.78 |
| **c-KIT** | **1** | -9.35 | O18 | NZ-LYS593 | HBA | 2.16 |
|  | **2** | -8.69 | O17 | N-CYS673 | HBA | 2.17 |
|  | **3** | -12.62 | O19  O17 | O-CYS673  NZ-LYS593 | HBD  HBA | 2.00  2.37 |
|  | **5** | -16.31 | O16  O18  6-ring | OG1-THR670  N-CYS673  CD2-LEU595 | HBD  HBA  π-H | 2.32  2.48  3.26 |
|  | **7** | -13.91 | O28  O17  6-ring | O-HOH289  N-CYS673  CG1-VAL603 | HBD  HBA  π-H | 1.93  1.91  3.05 |
|  | **12** | -13.549 | O11  6-ring | N-CYS673  CG1-VAL603 | HBA  π-H | 1.87  2.90 |
|  | **Sunitinib** | -11.48 | N7  O3  6-ring | GLU671  N-CYS673  CG1-VAL603 | HBD  HBA  π-H | 2.27  1.99  2.93 |
| **IGFR1** | **1** | -8.43 | O18 | N-MET1082 | HBA | 1.74 |
|  | **2** | -8.10 | O17 | N-MET1082 | HBA | 1.93 |
|  | **3** | -9.68 | O17  O18 | N-MET1082  O-LEU1005 | HBA  HBD | 2.26  2.01 |
|  | **5** | -11.04 | O17  O17 | N-SER1009  NZ-LYS1033 | HBA  HBA | 2.02  1.83 |
|  | **7** | -12.64 | O17 | N-MET1082 | HBA | 2.38 |
|  | **12** | -9.62 | O11  6-ring | N-MET1082  CB-LEU1005 | HBA  π-H | 1.75  2.72 |
|  | **741** | -12.98 | S25  6-ring | O-GLY1152  CG-MET1079 | HBD  π-H | 2.75  3.13 |
|  | **ibutamoren mesylate** | -9.98 | O5 | N-MET1082 | HBA | 3.26 |
| **PTEN** | **1** | 6.05 | O18  O18  O18 | N-LYS125  NE-ARG130  NH2-ARG130 | HBA  HBA  HBA | 2.58  2.18  2.19 |
|  | **2** | -8.45 | O17 | N-GLY129 | HBA | 2.07 |
|  | **3** | -9.57 | O17 | N-GLY129 | HBA | 2.44 |
|  | **5** | -10.42 | O19  O18  O21 | SG-CYS124  NE2-HIS93  NE2-GLN171 | HBD  HBA  HBA | 3.39  1.84  2.417 |
|  | **7** | -8.97 | O18  O27  O28  C23 | NE2-HIS93  N-HIS93  N-GLY129  5-ring HIS93 | HBA  HBA  HBA  π-H | 2.59  1.83  1.87  2.79 |
|  | **12** | -11.89 | O14 | NE-ARG130 | HBA | 2.14 |
|  | **TLA** | -14.18 | O1  O11  O11  O2  O4  O41  O41  O4  O4 | OG1-THR131  N-LYS128  N-THR131  N-ALA126  N-HIS93  N-HIS93  NE2-GLN171  NE-ARG130  NH2-ARG130 | HBA  HBA  HBA  HBA  HBA  HBA  HBA  Ionic  Ionic | 3.14  2.41  3.47  3.16  2.89  3.19  2.98  2.95  3.578 |
| **PI3K** | **1** | -9.41 | O18 | N-ASP933 | HBA | 3.10 |
|  | **2** | -8.59 | O17 | N-VAL851 | HBA | 2.08 |
|  | **3** | -10.55 | O17 | N-ASP933 | HBA | 1.81 |
|  | **5** | -13.11 | O18  O21 | N-ASP933  N-VAL851 | HBA  HBA | 2.02  1.88 |
|  | **6** | -12.96 | O29  O17 | O-SER854  N-ASP933 | HBD  HBA | 2.14  2.17 |
|  | **12** | -9.41 | O11 | N-VAL851 | HBA | 2.33 |
|  | **Apitolisib** | -12.06 | O3  O4  N5  N8 | NZ-LYS802  NZ-LYS802  O-HOH1203  N-VAL851 | HBA  HBA  HBA  HBA | 2.50  1.90  2.90  2.77 |
|  | **Leniolisib** | -10.94 | F3  O4  O4 | NZ-LYS802  N-SER854  OG-SER854 | HBA  HBA  HBA | 2.29  2.61  2.61 |
|  | **Samotolisib** | -14.13 | O1  O2 | O-HOH1203  NZ-LYS802 | HBA  HBA | 2.54  2.37 |
|  | Quinazoline-Derivative | -6.39 | N31  N44  N50  O51 | N-VAL851  O-HOH1203  NZ-LYS802  NZ-LYS802 | HBA  HBA  HBA  HBA | 2.22  2.28  2.60  2.43 |
| **Notch 1** | **1** | -8.29 | O18 | NH1-ARG1599 | HBD | 2.22 |
|  | **2** | -8.52 | O17 | NE2-GLN1528 | HBA | 1.96 |
|  | **3** | -10.31 | O17 | NE2-GLN1528 | HBA | 1.83 |
|  | **5** | -14.69 | O17 | N-THR94 | HBA | 1.78 |
|  | **7** | -11.58 | O15  O27  O17  O29 | OD1-ASP1521  O-SER1502  NE2-HIS1602  NE1-TRP100 | HBD  HBD  HBA  HBA | 2.74  2.60  2.27  2.57 |
|  | **12** | -10.51 | O11 | NZ-LYS1538 | HBA | 2.00 |
|  | **Crenigacestat** | -10.26 | N11  O5 | OE1-GLU1526  NH1-ARG1599 | HBD  HBA | 2.12  2.35 |
| **CDK4** | **1** | -1.18 | O18 | N-ALA182 | HBA | 2.01 |
|  | **2** | -2.87 | O17 | NZ-LYS142 | HBA | 2.40 |
|  | **3** | -0.54 | O17 | NH2-ARG139 | HBA | 2.50 |
|  | **5** | -8.05 | O17 | NH2-ARG139 | HBA | 1.81 |
|  | **7** | -6.22 | O30  O17  O29 | SD-MET169  N-ALA182  NH2-ARG181 | HBD  HBA  HBA | 3.14  1.83  1.73 |
|  | **12** | -8.23 | O11 | NH2-ARG139 | HBA | 1.86 |
|  | **Omacetaxine mepesuccinate** | -5.39 | O9  O9 | NE-ARG139  NH2-ARG139 | HBA  HBA | 2.30  2.99 |
| **PARP1** | **1** | -10.14 | O32 | N-TYR896 | HBA | 2.19 |
|  | **2** | -9.56 | O37 | N-GLY863 | HBA | 2.41 |
|  | **3** | -10.32 | O35  C10 | N-GLY863  6-ring TYR896 | HBA  π-H | 2.17  3.12 |
|  | **5** | -14.64 | O27  O32 | OE2-GLU988  OG-SER904 | HBD  HBD | 2.23  1.96 |
|  | **7** | -18.74 | O20  O27 | OE2-GLU988  O-HOH1216 | HBD  HBA | 1.97  2.42 |
|  | **12** | -13.74 | O14 | N-TYR896 | HBA | 1.79 |
|  | **Niraparib** | -13.18 | N42  O41  C15  5-ring | O-GLY863  N-GLY863  6-ring TYR896  6-ring TYR907 | HBD  HBA  π-H  π-π | 2.04  2.07  2.95  2.96 |
| **CA-II** | **1** | -10.75 | O32  O32  O32  O32 | OG1-THR200  O-HOH401  O-HOH464  O-HOH483 | HBA  HBA  HBA  HBA | 2.23  2.78  2.31  2.26 |
|  | **2** | -9.64 | O37  O37 | ND2-ASN67  O-HOH524 | HBA  HBA | 2.02  2.30 |
|  | **3** | -10.39 | O26  O26  O35  O39 | OE2-GLU69  ND2-ASN67  O-HOH608  O-HOH611 | HBD  HBA  HBA  HBA | 2.58  2.77  2.65  2.58 |
|  | **5** | -21.98 | O27  O32  6-ring | OG1- THR200  N-THR199  CD2-LEU198 | HBD  HBA  π-H | 2.34  2.04  3.14 |
|  | **7** | - 3.26 | 6-ring | 6-ring PHE 131 | π-π | 2.99 |
|  | **12** | -13.88 | O14  O14  6-ring | N-THR199  OG1-THR199  OG1-THR200 | HBA  HBA  π-H | 1.45  1.74 2.88 |
|  | **Acetazolamide** | 13.87 | O13  O19  O19 | HOH524  OG1-THR199  ZN-302 | HBA  HBA  Metal | 2.87  2.76  2.63 |

HBA = Hydrogen Bond Acceptor, HBD = Hydrogen Bond Donor, hydrophobic interactions = π-H

π-π

**Figure S1**: ^1^H, ^13^C-NMR and ESI-MS spectra of the known compounds (**1**-**20**).

ESI-MS of compound **1**

^1^H-NMR (600 MHz, CD_3_OD) Spectrum of compound **1**

^13^C-NMR (125 MHz, CD_3_OD) Spectrum of compound **1**

ESI-MS of compound **2**

^1^H-NMR (600 MHz, CD_3_OD) Spectrum of compound **2**

^13^C-NMR (125 MHz, CD_3_OD) Spectrum of compound **2**

ESI-MS of compound **3**

^1^H-NMR (600 MHz, CD_3_OD) Spectrum of compound **3**

^13^C-NMR (125 MHz, CD_3_OD) Spectrum of compound **3**

ESI-MS of compound **4**

^1^H-NMR (600 MHz, CD_3_OD) Spectrum of compound **4**

 ^13^C-NMR (125 MHz, CD_3_OD) Spectrum of compound **4**

ESI-MS of compound **5**

^1^H-NMR (600 MHz, CD_3_OD) Spectrum of compound **5**

^13^C-NMR (125 MHz, CD_3_OD) Spectrum of compound **5**

ESI-MS of compound **6**

^1^H-NMR (600 MHz, CD_3_OD) Spectrum of compound **6**

^13^C-NMR (125 MHz, CD_3_OD) Spectrum of compound **6**

ESI-MS of compound **7**

^1^H-NMR (600 MHz, CD_3_OD) Spectrum of compound **7**

^13^C-NMR (125 MHz, CD_3_OD) Spectrum of compound **7**

ESI-MS of compound **8**

^1^H-NMR (600 MHz, CD_3_OD) Spectrum of compound **8**

^13^C-NMR (125 MHz, CD_3_OD) Spectrum of compound **8**

ESI-MS of compound **9**

**Figure S26**: ^1^H-NMR (600 MHz, CD_3_OD) Spectrum of compound **9**

^13^C-NMR (125 MHz, CD_3_OD) Spectrum of compound **9**

ESI-MS of compound **10**

^1^H-NMR (600 MHz, CD_3_OD) Spectrum of compound **10**

^13^C-NMR (125 MHz, CD_3_OD) Spectrum of compound **10**

ESI-MS of compound **11**

^1^H-NMR (600 MHz, CD_3_OD) Spectrum of compound **11**

^13^C-NMR (125 MHz, CD_3_OD) Spectrum of compound **11**

ESI-MS of compound **12**

^1^H-NMR (600 MHz, DMSO) Spectrum of compound **12**

^13^C-NMR (125 MHz, DMSO) Spectrum of compound **12**

ESI-MS of compound **13**

^1^H-NMR (600 MHz, CD_3_OD) Spectrum of compound **13**

^13^C-NMR (125 MHz, CD_3_OD) Spectrum of compound **13**

ESI-MS of compound **14**

^1^H-NMR (600 MHz, CD_3_OD) Spectrum of compound **14**

^13^C-NMR (125 MHz, CD_3_OD) Spectrum of compound **14**

ESI-MS of compound **15**

^1^H-NMR (600 MHz, CD_3_OD) Spectrum of compound **15**

^13^C-NMR (125 MHz, CD_3_OD) Spectrum of compound **15**

ESI-MS of compound **16**

^1^H-NMR (600 MHz, CDCl_3_) Spectrum of compound **16**

^13^C-NMR (125 MHz, CDCl_3_) Spectrum of compound **16**

^1^H-NMR (600 MHz, CD_3_OD) Spectrum of compound **17**

^13^C-NMR (125 MHz, CD_3_OD) Spectrum of compound **17**

ESI-MS of compound **18**

^1^H-NMR (600 MHz, CDCl_3_) Spectrum of compound **18**

^13^C-NMR (125 MHz, CDCl_3_) Spectrum of compound **18**

ESI-MS of compound **19**

^1^H-NMR (600 MHz, CDCl_3_) Spectrum of compound **19**

^13^C-NMR (125 MHz, CDCl_3_) Spectrum of compound **19**

^1^H-NMR (600 MHz, CDCl_3_) Spectrum of compound **20**

^13^C-NMR (125 MHz, CDCl_3_) Spectrum of compound **20**
